# The association between fear of malpractice and burnout among Chinese medical workers: The mediating role of legal consciousness

**DOI:** 10.1186/s12888-022-04009-8

**Published:** 2022-05-25

**Authors:** Fei Liang, Shu Hu, Youqi Guo

**Affiliations:** 1 Department of Histology and Embryology, College of Basic Medicine, China Medical University, Shenyang, People’s Republic of China; 2 College of Marxism, China Medical University, No.77 Puhe Road, Shenyang North New Area, Shenyang City, Liaoning Province People’s Republic of China

**Keywords:** Fear of malpractice, Burnout, Legal consciousness, Chinese medical worker

## Abstract

**Background:**

As a major reason for defensive medicine, the status and effect of “fear of malpractice” among Chinese medical staff is an important topic that needs to be studied. Our study investigated fear of malpractice among Chinese medical workers, assessed its association with burnout, and explored the mediating role of legal consciousness between these factors.

**Design:**

A quantitative, cross-sectional study.

**Setting:**

All respondents were investigated using a self-report questionnaire. Demographic characteristics and measurements including a fear of malpractice scale, and a burnout and legal consciousness scale were employed. The effect of fear of malpractice on burnout was examined by carrying out a binary regression analysis. A mediation model was used to test the mediating role of legal consciousness.

**Participants:**

The study sample included 1031 doctors and nurses (297 male and 734 female; age = 36.3 ± 8.98).

**Results:**

The average score of fear of malpractice was 20.97 ± 5.34. Respondents with higher levels of fear of malpractice were more prone to burnout ([OR] = 2.865; 95% CI 1.942–4.226). Legal consciousness partially and negatively mediated the effect of fear of malpractice on burnout.

**Conclusion:**

High levels of fear of malpractice were found among Chinese medical workers, and this fear had a significant effect on burnout. Legal consciousness may be a resource that can alleviate burnout.

## Background

Fear of malpractice was considered as the main reason for defensive medicine, the latter of which refers to “practice based primarily on the fear of litigation rather than on expected patient outcomes” [1, 2]. Fear of malpractice not only resulted in excessive medical expenditure [3], but also became an important obstacle to compliance with medical guidelines [4]. However, the study showed that Chinese physicians broadly accepted defensive medicine [5]. It may be due to misaligned incentives in the Chinese health system, which encourage medical workers to practice overprescription as a means of supplementing their low incomes [6]. Some researchers have argued that Chinese medical workers exaggerate the actual risks that they really face [7]. For example, medical malpractice lawsuits are relatively rare in China. In 2014, 115,000 medical disputes were recorded in China, and only 17% of medical litigation cases were accepted by the court [8]. However, with a frequent incidence of workplace violence in hospitals [9], many Chinese medical workers have tend to regard patients as a genuine threat, and therefore, fear of malpractice is not merely an excuse for “economically motivated corruption” [3]. The low medical litigation rate also implies that Chinese medical workers lack sufficient legal resources to deal with medical disputes. As such, they try to avoid any involvement in them. Psychologically, this manifests as fear of malpractice. Previous studies that have examined fear of malpractice primarily focused on defensive medicine as practiced by emergency physicians [10–14], and fear of malpractice as a mental status among Chinese medical workers needs further investigation.

Burnout is an individual psychological syndrome [15], with high prevalence among Chinese medical workers [16–19]. In addition to adverse psychological consequences such as anxiety and depression [20], burnout also has physical consequences that may manifest in the form of an increased risk of cardiovascular diseases, type 2 diabetes, and all-cause mortality [21–23]. The burnout among medical workers is a noteworthy phenomenon, as it threatens not only the personal health of medical staff members, but also that of their patients [24]. The common conceptual framework of burnout is the Maslach Burnout Inventory (MBI) [25]. In this framework, burnout can be divided into three dimensions: emotional exhaustion (EE) or exhaustion, depersonalization (DP) or cynicism, and reduced personal accomplishment (rPA) or professional efficacy. The MBI is often described in terms of a sequential conceptual model which identifies the following: emotional exhaustion (EE) that firstly occurs in response to job-related stressors such as overload, which are followed by detachment reactions (DP or cynicism), and finally, by simultaneously or sequentially reduced PA [15, 26]. It supports the general notion of burnout that “burnout is a prolonged response to chronic job stressors” [15].

Previous studies have shown that malpractice lawsuits are an important stressor for physicians [27], and a significant correlation has been found between fear of litigation and psychological disorders such as depression, anxiety, and stress among expatriate nurses in Saudi Arabia [28]. As an important job stressor for medical workers, it is reasonable to hypothesize that the fear of malpractice may lead to burnout.

Aside from the sequential conceptual model, another important burnout models is the Job Demands-Resources (JD-R) model which based on the demands-resources imbalance theory [29]. Bakker et al. in 2021 integrated a self-regulation framework into JD-R model to explain burnout [30]. The JD-R model holds that high job demands and low job resources may cause job strain and eventually lead to burnout, workers may actively use their personal resources to cope with job demands. Whereas job resources can fulfill psychological demands and buffer the influence of job demands on burnout. Moreover, workers can actively adopt self-regulation strategies to influence their own job characteristics; that is, they may optimize their job demands and resources through job crafting (adaptive self-regulation) or undermine their own functioning at work (maladaptive self-regulation) [30]. According to the JD-R model, the fear of malpractice could cause medical workers actively use personal psychological legal resources to cope with this emotional demand, therefore, prompted us to examine the issue of legal consciousness among Chinese medical workers.

**Fig. 1:**
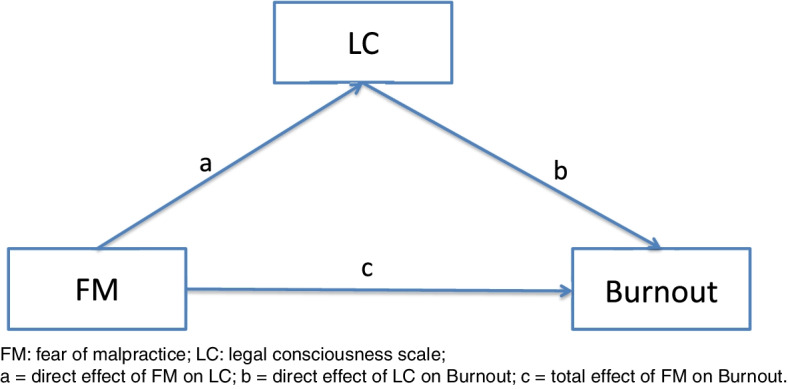
The mediating role of LC on the relationship between FM and Burnout

Legal consciousness is a broad and vague concept concerning an individual’s awareness and understanding of the law and legal rights [31]. Horák et al. defined that legal consciousness is “a complex of law-related knowledge, skills, attitudes, beliefs, and values of an individual” [32]. In previous literature publications, “legal consciousness” was used to describe individuals’ perceptions of the role of the law in sexual harassment [31], sexual assaults [33] and LGBT [34]. In the current study, legal consciousness is a personal psychological resource that can help an individual to deal with fear of malpractice, and it refers to medical workers’ attitude to study and apply legal knowledge. According to JD-R theory, fear of malpractice could prompt medical workers actively study and apply legal knowledge to cope with this emotional demand, thus, expanding their legal consciousness. Furthermore, legal consciousness also can affect burnout independently. Consequently, legal consciousness may buffer the effect of fear of malpractice on burnout. Therefore, we hypothesized that fear of malpractice is a predictor for burnout, and legal consciousness could be a negative mediating factor between fear of malpractice and burnout (Fig. 1). Hence, the objectives of this study were to: 1) investigate the status of fear of malpractice among Chinese medical staff andevaluate its related factors; 2) assess the effect of fear of malpractice on burnout; 3) and examine legal consciousness as a mediator between fear of malpractice and burnout.

## Methods

### Study design and participants

A cross-sectional study was conducted from September 2017 to January 2018. According to the geographical distribution, we selected three cities in Liaoning Province, China, and 2–3 general tertiary hospitals from each city. Doctors and nurses from a total of eight hospitals were invited to participate in the survey. We received assistance from the administrators and medical workers in all hospitals during the questionnaire survey. In most cases, questionnaires were distributed and collected on the spot. A total of 1200 questionnaires were distributed among these medical workers. After excluding questionnaires with missing data, 1031 valid questionnaires were included in this study.

This study was approved by the Committee on Human Experimentation of China Medical University (No. cmu2015079).

### Demographic characteristics

In this study, demographic characteristics included gender, age (< 30/30–40/ > 40), marital status (married/ single, divorced or widowed), education (college degree or lower/bachelor degree/Master degree or higher), and position (doctor or nurse).

### Measurements

#### Fear of malpractice

We used the Chinese version of the Fear of Malpractice (FM) Scale which includes six items scored on a 5-point Likert scale ranging from 1 “strongly disagree” to 5 “strongly agree.” The FM scale was first developed by Katz et al. [10]. The Cronbach’s alpha value for the FM Scale in this study was 0.839, indicating acceptable reliability. The FM was categorized by referring to a previous study [35]. Specifically, the sum of responses was calculated and converted to a 1- to 100-point scale, and categorized based on their observed distribution into tertiles (Low, Medium, and High): Low FM group (*N* = 346, 33.6%), Medium FM group (*N* = 311, 30.2%)) and High FM group (*N* = 374, 36.3%).

#### Burnout

A standardized Chinese version of the Maslach Burnout Inventory-Human Service Survey (MBI-HSS) was used. The MBI-HSS consists of 22 items scored according to a 7-point Likert-type scale ranging from 0 (never) to 6 (every day). It contains three subscales: emotional exhaustion (EE, nine items), depersonalization (DP, five items), and personal accomplishment (PA, eight items). The cutoff point for the high levels of each subscale was EE ≥ 27, DP ≥ 13, and PA ≤ 31, respectively [36–38]. In this study, medical workers who had high scores on all three subscales were classified as suffering from burnout. The Cronbach’s alpha values for the MBI-HSS, EE, DP and PA was 0.836, 0.878, 0.787 and 0.867, respectively. In further analysis, a weighted burnout score was introduced to convert the MBI-HSS into a continuous variable. This strategy was adopted by a previous study [39], and is similar to the strategy used in the Maslach Burnout Inventory-General Survey (MBI-GS) [19, 40]. All items for PA were reverse coded before calculation (rPA). The equation was defined as burnout = 0.4XEE + 0.3XDP + 0.3XrPA.

#### Legal Consciousness Scale (LC)

A self-developed scale consisting of five items was used to investigate Chinese medical workers’ legal consciousness (Table 3). For examples, the items included questions such as “Do you think that the legal knowledge you possess is helpful for your treatment and diagnosis of medical issues?” Items were scored using a 4-point Likert-type scale ranging from 0 to 3 (none to always). The validation of LC was carried out by means of exploratory factor analysis (EFA) and confirmatory factor analysis (CFA). The Cronbach’s alpha value of LC was 0.817 in this study.

SPSS AMOS software version 23 was used to conduct a confirmatory factor analysis of the LC. Model fit indices included: the Chi-square statistic and associated probability, Comparative Fit Index (CFI), Tucker Lewis Index (TLI), Standardized Root Mean Square Residual (SRMR), and Root Mean Square Error of Approximation (RMSEA). The acceptable values for model fit indices were CFI ≥ 0.90, TFI ≥ 0.90, SRMR ≤ 0.05, and RMSEA ≤ 0.10 [41].

### *S*tatistical analysis

All data from the questionnaires were input in EpiData 3.0 software. Statistical analyses were performed by SPSS version 25.0 (IBM, Armonk, NY, USA). All statistical tests were two-tailed, and *p* < 0.05 was deemed as significant.

Student’s *t* test and a one-way analysis of variance (ANOVA) were used to examine differences in the FM scores among groups. In the case of data with uneven variance, the Kruskal–Wallis nonparametric test was performed. FM scores were treated as continuous variables. Correlations between FM, burnout, and LC were examined by a Pearson test.

A binary regression was used to identify the effect of FM on burnout, and burnout was used as the criterion variable. Demographic factors and FM were considered as potential risk factors. We adopted a forward stepwise likelihood ratio elimination model. Finally, age, marital status and FM were included in the model.

To verify the mediation model, we used a macro program, PROCESS 3.41 program for SPSS. We used Model 4 of PROCESS macro. Burnout and its subscales (EE, DP and rPA) were Y variables, FM was the X variable, and LC was the mediator. The path was controlled for gender and marital status; the displayed effects are standardized. In this model, the bootstrap confidence interval of parameter estimation was obtained. If the confidence interval (CI) did not include zero, the result was considered significant.

## Results

### The characteristics of the respondents and FM

The characteristics of the respondents are shown in Tables 1 and 2. A total of 625 doctors and 406 nurses participated in this study. Most of the respondents were female (71.2%), 45.7% of respondents were between 30 and 39 years old, and 24.3% were younger than 30 years old. About 71.2% of the respondents were married. Approximately half of respondents held a bachelor degree. Among all respondents, 211 (20.5%) had burnout symptoms.

**Table 1:** Characteristics of the study population (*N* = 1031)

Factors	*N* = 1031 (%)
Gender	
Male	297 (28.8%)
Female	734 (71.2%)
Age(y)	
< 30	251 (24.3%)
30–39	471 (45.7%)
≥ 40	309 (30.0%)
Marital status	
Married	734 (71.2%)
Single/ Divorced /widowed	297 (28.8%)
Education	
Junior college course or lower	245 (23.8%)
Bachelor degree	521 (50.5%)
Master’s degree or higher	265 (25.7%)
Position	
Doctor	625 (60.6%)
Nurse	406 (39.4%)
Burnout	
Yes	211 (20.5%)
No	820 (79.5%)
FM	
Low	346 (33.6%)
Medium	311 (30.2%)
High	374 (36.3%)

*FM* Fear of Malpractice

**Table 2:** The distribution and FM scores among the study population (*N* = 1031)

**Factors**	**FM (Low)** ***N* = 346 (%)**	**FM (Medium)** ***N* = 311 (%)**	**FM (High)** ***N* = 374 (%)**	**FM scores**	***F***	***P***
Gender					**1.508**	**0.149**
**Male**	85 (24.6%)	88 (28.3%)	124 (33.2%)	21.35 ± 5.59		
**Female**	261 (75.4%)	223 (71.7%)	250 (66.8%)	20.82 ± 5.23		
Age(y)*					5.220*	**0.022**.^**1**^
** < 30**	106 (30.6%)	64 (20.6%)	81 (21.7%)	20.03 ± 5.79		
**30–39**	149 (43.1%)	141 (45.3%)	181 (48.4%)	21.30 ± 5.22		
** ≥ 40**	91 (26.3%)	106 (34.1%)	112 (29.9%)	21.24 ± 5.05		
Marital status*					16.463**	**0.018**
**Married**	227 (65.6%)	234 (75.2%)	273 (73.0%)	21.24 ± 50.1		
**Single/ Divorced /widowed**	119 (34.4%)	77 (24.8%)	101 (27.0%)	20.30 ± 6.02		
Education					**1.977**	**0.139**
**Junior college course or lower**	102 (29.5%)	67 (21.5%)	76 (20.3%)	20.39 ± 5.12		
**Bachelor degree**	165 (47.7%)	160 (51.4%)	196 (52.4%)	21.10 ± 5.48		
**Master’s degree or higher**	79 (22.8%)	84 (27.0%)	102 (27.3%)	21.24 ± 5.22		
Position					**1.738**	**0.791**
**Doctor**	204 (59.0%)	189 (60.8%)	232 (62.0%)	20.93 ± 5.50		
**Nurse**	142 (41.0%)	122 (39.2%)	142 (38.0%)	20.02 ± 5.09		
Burnout**					6.241*	**0.000**
**Yes**	45 (13.0%)	55 (17.7%)	111 (29.7%)	23.00 ± 4.6		
**No**	301 (87.0%)	256 (82.3%)	263 (70.3%)	20.45 ± 5.39		

1 The differences were examine by Student’s *t* test and ANOVA. In the case of data with uneven variance, the Kruskal–Wallis nonparametric test was performed

2.^1^ Perform Kruskal–Wallis test

3 The Kruskal–Wallis test for Age showed the significant difference between < 30 and other groups, but had no significant difference between 30–39 and ≥ 40 group

^**^: *P* < 0.01. *: *P* < 0.05.1

The mean score of FM was 20.97 ± 5.34. Demographic characteristics in different FM groups are illustrated in Table 2. The Student’s t test and ANOVA test showed that the FM scores were significantly different in respect to age and marital status, but no significant difference was observed in the case of gender, and education and job position. The results showed medical workers who were married and over 30 years of age were likely to have high FM scores.

### The Validation of the LC

The results of the EFA and the distribution of respondents for LC are detailed in Table 3. In the EFA, we used Varimax rotation with Kaiser Normalization and the Principal Axis Factoring extraction method. The value of the KMO test was 0.834 and the Bartlett sphericity test was significant (*p* < 0.001). All six items belong to single extracted factor 1. The factorial loads of all of the items were greater than 0.7. Factor 1 had a saturation of 58.65% of the total variance. In this study, the Cronbach’s alpha coefficient for the LC was 0.817, which indicated that the LC had a high degree of internal consistency.

**Table 3:** The distribution and exploratory factor analysis for the Legal Consciousness Scale

Item	N (%)				Factor 1
	None	Sometimes	Often	Always	
Q1 Do you think the legal knowledge you have is helpful for your medical treatment and diagnosis?	25 (2.4)	107 (10.4)	339 (32.9)	560 (54.3)	0.803
Q2 Do you think the legal knowledge you have is helpful to prevent medical dispute with patients	58 (5.6)	125 (12.1)	380 (36.9)	468 (45.4)	0.765
Q3 Do you think that once there is a medical dispute, you can take the initiative to seek legal protection?	43 (4.2)	178 (17.3)	341 (33.1)	469 (45.5)	0.722
Q4 Do you think you have enough legal knowledge or do you always think you need to study the legal knowledge?	27 (2.6)	139 (13.5)	421 (40.8)	444 (43.1)	0.713
Q5 Do you think it is necessary for you to enhance legal literacy?	17 (1.6)	53 (5.1)	372 (36.1)	589 (57.1)	0.820

The values ​​of the Kaiser–Meyer–Olkin (KMO) test was 0.834 (satisfactory values ​​ > 0.500) and the Bartlett sphericity test was significant (*p* < 0.001). All of the items’ factorial loads were > 0.7

**Fig. 2:**
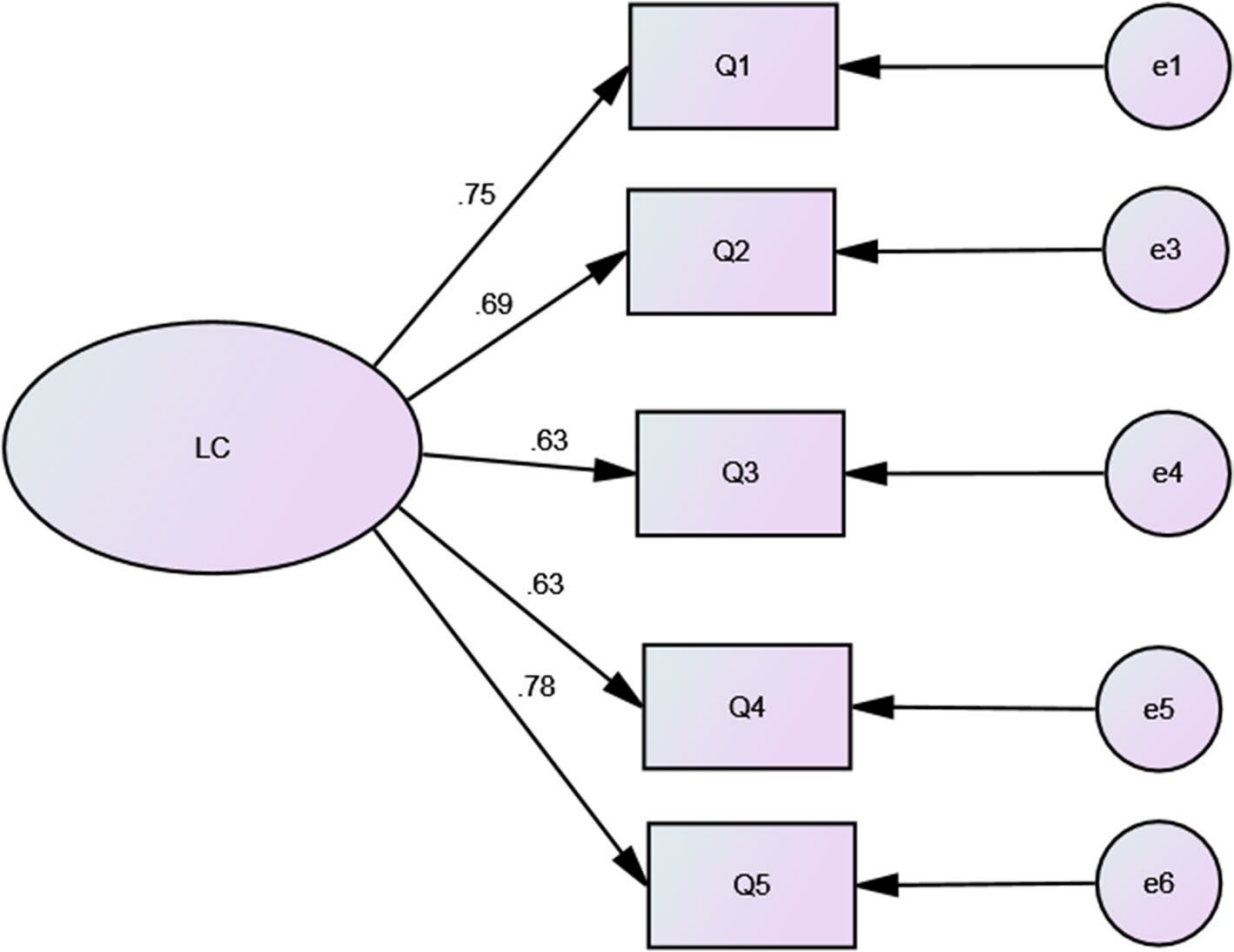
: Confirmatory factor analysis for the Legal Consciousness Scale. The confirmatory factor analysis for LC showed: χ2 = 46.0, *p* < 0.001; SRMR = 0.0288, GFI = 0.982, NFI = 0.973, CFI = 0.976, RMSEA = 0.089

The results of the CFA are shown in Fig. 2. The model fit indices for the LC were as follows: χ2 = 46.0, *p* < 0.001; SRMR = 0.0288, GFI = 0.982, NFI = 0.973, CFI = 0.976, RMSEA = 0.089. These results showed that the LC is an acceptable scale for legal consciousness.

### The binary regression analysis of FM for burnout

The binary regression analysis of FM for burnout is shown in Table 4. Finally, age, marital status and FM were included in the logistic model. Compared with the Low FM group, respondents in the High FM group were significantly prone to burnout (odds ratio [OR] = 2.865; 95%CI, 1.942–4.226). This indicated that FM was a predicator of burnout.

**Table 4:** Binary regression analysis of FM on burnout

Factor	Burnout			
	OR	95% CI		*P*
Gender				
Male	1.553	1.117	2.159	0.009
Female(ref)				
Marital status				
Married	1.587	1.136	2.218	0.007
Single/ Divorced /widowed (ref)				
FM				
Low (ref)				
Medium	1.493	0.969	2.300	0.069
High	2.865	1.942	4.226	0.000

A forward stepwise likelihood ratio elimination model was adopted. Burnout was used as the criterion variable, demographic factors and FM were considered as potential risk factors. Finally, age, marital status and FM were included in the model

### The correlation between the FM, LC and burnout

Table 5 shows the correlation between the FM, LC, burnout and its subscales. The FM scale was positively associated with LC (*r* = 0.115, *P* < 0.01) and burnout (*r* = 0.324, *P* < 0.01), whereas the LC scale was negatively associated with burnout (*r* =  − 0.187, *P* < 0.01) and its subscales (EE *r* =  − 0.060, *P* < 0.01; DP *r* =  − 0.128 *P* < 0.01; rPA *r* =  − 0.289, *P* < 0.01).

**Table 5:** Correlations between FM, LC and burnout

**Variables**	**Mean**	**SD**	1	2	3	4	5
1. FM	20.97	5.34	1				
2. LC	11.54	3.02	0.115**	1			
3. EE	24.06	10.89	0.415**	-0.060*	1		
4. DP	10.35	6.19	0.326**	-0.128**	0.775**	1	
5.rPA	25.47	8.95	-0.063*	-.0.289**	0.119**	0.244**	1
6.Burnout	2.65	0.93	0.324**	-0.187**	0.874**	0.892**	0.521**

The Pearson correlation test were performed

*FM* Fear of Malpractice, *LC* Legal Consciousness Scale, *EE* Emotional Exhaustion, *DP* Depersonalization, *rPA* reduced Personal Accomplishment

^**^ : *P* < 0.01. ^*^ : *P* < 0.05

### LC as a mediator between the FM and burnout

We hypothesized that LC may have a potential mediating effect between FM and burnout (Fig. 1). The mediation effect analysis is shown in Table 6. This illustrates that FM had a significantly positive effect on LC (a = 0.1158 *p* < 0.01), and LC had a significant negative effect on burnout (b = -0.2285 *p* < 0.01), indicating that LC played a mediating role between FM and burnout. The mediation effect of LC for burnout was -0.0265 (95% CI: -0.0448 ~ -0.0103). In respect to the direct effect between FM and burnout, the percentage of the mediation effect that was mediated by LC was 7.45%. This indicated that LC partially negatively mediated the relationship between FM and burnout.

**Table 6:** Results of mediation analyses (standardized)

Paths	a	b	c’	a*b	95%CI of a*b (LL and UL)		C	SE	R.^2^
FM → LC → Burnout	0.1158**	-0.2285**	0.3558**	-0.0265*	**-0.0448**	**-0.0103**	0.3293**	**0.0087**	**0.1091**
FM → LC → EE	0.1158**	-0.1090**	0.4269**	-0.0126*	**-0.0243**	**-0.0040**	0.4143**	**0.0052**	**0.1719**
FM → LC → DP	0.1158**	-0.1694**	0.3504**	-0.0196*	**-0.0349**	**-0.0073**	0.3308**	**0.0070**	**0.1096**
FM → LC → rPA	0.1158**	-0.2880**	**-0.0521**	-0.0333*	**-0.0576**	**-0.0134**	**-0.0521**	**0.0110**	**0.0195**

*N* = 1031; The path was controlled for gender and marital status; the displayed effects are standardized; *FM* Fear of Malpractice, *LC* Legal awareness scale, *EE* Emotional Exhaustion, *DP* Depersonalization, *rPA* reduced Personal Accomplishment

^**^statistical significance level of *P* < 0.01, the indirect effect is significant (*) when the 95% CI does not include 0; SE, bootstrap regression standard error; R^2^, variance accounted for;

a = direct effect of FM on LC; b = direct effect of LC on Burnout; c = total effect of FM on Burnout; c’ = direct effect of FM on Burnout; a*b = indirect effect on Burnout

## Discussion

In this study, we carried out a cross-sectional survey to investigate the status of fear of malpractice among Chinese medical workers, analyzed the association between fear of malpractice and burnout, and the mediating role of legal consciousness. Our study has an important implication in the fear of malpractice knowledge field and is a supplement of JD-R burnout model.

Although fear of malpractice (FM) plays an important role in defensive medical practice, studies on the subject are still lacking. To our knowledge, with the exception of a Chinese literature publication in 2018 [42], our group is the first to examine FM among Chinese medical workers. The mean score of FM was 20.89 in this study, which was higher than the midpoint of FM scale (FM = 18). In our survey, 66.4% medical workers had FM scores over the midpoint, which indicated that many Chinese medidcal workers genuinely experience fears in relation to their work. Incidents of workplace violence, even criminal workplace violence, in health facilities in China have been widespread [9, 43, 44]. The types of violence reported include burning mock paper money, placing a corpse in the hospital, hanging banners, blocking hospital gates and smashing hospital property [44]. Therefore, individuals will try to avoid medical disputes, which manifests as fear of malpractice.

This study showed that medical workers who were married and over 30 years of age tended to experience high levels of fear regarding malpractice, and scored high on the FM. However, the differences among most demographic characteristics were slight, which implies that fear of malpractice is a common psychological issue observed among Chinese medical workers. Doctors, in particular, did not score significantly higher than nurses, although doctors assume greater legal responsibility. This result indicated that both doctors and nurses try to avoid medical disputes, and both doctors and nurses perceive threats in relation to their work.

Burnout is a common psychological problem that is observed among Chinese medical workers [16–19, 45]. However, the assessment for burnout outlined in medical literature publications has been inconsistent [46]. Among three MBI dimensions, EE is often regarded as the core syndrome of burnout, and it has sometimes been taken as an independent measure of burnout [47]. However, study shows that EE alone does not seem to be a sufficient proxy for burnout, and DP may be more of a core part of burnout than EE [48]. Some researchers argue that the conceptualization of burnout should consist of at least EE and DP and it should also include rPA [49]. In 2020, Schaufeli et al. established a new cluster conceptual framework that included four core dimensions (exhaustion, mental distance, and impaired emotional and cognitive impairment) and three secondary dimensions (depressed mood, psychological distress, and psychosomatic complaints) [50]. This framework tackled some essential flaws in the MBI, but its validity and applicability still need to be evaluated in the future. Although manner in which burnout should be assessed is a matter of debate, a combination of the three dimensions is still the most frequently applied instrument to measure burnout [51]. The MBI is still the most widely applied measure and it is regarded as the gold standard of burnout assessment. We applied strict criteria that required high scores on all three subscales [51]. 29.7% of respondents in the High FM group reported burnout compared with 13.0% in the Low FM group. The binary regression analysis showed that those in the High FM group were prone to burnout ([OR] = 2.865), and FM can be regarded as a predicator for burnout. As a job-related stressor, fear of malpractice can contribute to an increased risk of burnout among medical workers. Alleviating fear of malpractice among medical workers should be an effective intervention to reduce burnout.

According to JD-R theory, when confronted with job-related stressors, medical workers may actively use their personal resources to cope with the situation. Thus, we focused on the issue of legal consciousness. As a concept in legal science, assessing legal consciousness is complicated, as it involves a cognitive component as well as emotional and behavioral elements [52]. Horák et al. reviewed 2,054 articles, included 156 relevant articles and identified six components of legal consciousness including general knowledge, skills, specific knowledge, attitudes, trust, and identity [32]. Although it is intricate, we only touched upon individual attitude to study and apply legal knowledge, which can be included in Horák et al. framework. To evaluate legal consciousness, we used a self-developed five-item legal consciousness scale (LC). The exploratory factor analysis and confirmatory factor analysis showed that the scale was acceptable. In our survey, most Chinese medical workers were willing to broaden their legal knowledge and apply it to their medical practice. The correlation analysis revealed a significant correlation between FM, LC and burnout. It is interesting that FM was significantly correlated with EE, whereas LC was only slightly correlated with EE. Discussions with Chinese medical workers revealed frequent complaints regarding fear of malpractice caused high exhaustion and the lack of protection afforded to them by Chinese law in the course of their practice of medicine. The correlation analysis supports the complaints among Chinese medical workers that fear of malpractice relates to higher levels of exhaustion, on which legal knowledge has only a slight effect.

We also analyzed the mediation model of LC in relation to FM and burnout. Although the Chinese medical workers often complained that the Chinese legal system, the results showed that LC alleviated their burnout. The mediation analysis revealed that LC partially negatively mediated about 7.45% of the direct effect of FM on burnout. These results verified the mediation model. In our study, FM had the most significant direct effect on EE, followed by DP, but had no significant effect on rPA. LC had the most significant negative effect on rPA, followed by DP, and had the least effect on EE. This is consistent with previous study which found that job demands were the most important predictors of EE, whereas job resources were the most important predictors of DP and PA [29]. When confronted with the threat of a medical dispute, medical workers are likely to broaden their legal knowledge to deal with or avoid potential disputes. The positive attitude of study and application of legal knowledge may also help medical workers to manage their exhaustion, approach the work, and experience a greater sense of personal accomplishment. Thus, the legal consciousness could be regard as a job resource that can help to alleviate burnout symptoms. Our study can help policy makers and hospital administrators to formulate effective interventions to reduce defensive medical practice and burnout among Chinese medical workers. The study proposes several suggestions. At a policy level, it is necessary to formulate more detailed regulations, clinical pathways and guidelines to clarify medical workers’ responsibilities and rights, which can provide a framework to guide their work and practice. Hospitals should be encouraged to resolve clinical disputes through the judicial system. At a management level, regular legal knowledge training should be provide to workers, and dedicated legal department should be charged with the task of providing institutionalized legal aid, which would connect medical workers with legal staff. To reduce burnout, personal and more robust security measures should also prove useful for hospital administrators.

However, it should be noted that decreasing the risk of malpractice may not be effective in the case of defensive medicine. For example, in five states in the US, which have the highest risk of malpractice risk, 68% of physicians practice defensive medicine. However, in the five states that have the lowest malpractice risk, 64% of physicians practice defensive medicine, which is only a marginal difference [1]. Adjusting misaligned incentives mechanisms and improving the relationship between medical workers and their patients with a view to reducing perceived threats could be the key to combatting defensive medicine.

Our study was carried out prior to the global COVID-19 pandemic. The reputation of Chinese medical workers has improved greatly during the COVID-19 pandemic. However, COVID-19 will not immediately or dramatically alter the medical situation. FM is still a common issue in the Chinese healthcare system. We believe that the COVID-19 outbreak will not change the conclusions of our study.

Our study was subject to a number of limitations. First, the data for the survey were obtained by using self-administrated questionnaires which may have produced self-report bias in the results. Second, the cross-sectional survey was not able to determine causal relationships. Third, as we mentioned above, the assessment of legal consciousness is complicated and our research is a preliminary study. Thus, the dimensions and assessment of legal consciousness need further exploration and verification. Finally, the results of the current study focused on the medical and legal environment in China, which means that the findings may not be easily generalizable to other countries.

In conclusion, our study investigated the status of fear of malpractice among Chinese medical workers, and verified the significant effect of FM on burnout. LC was found to play a mediating role in the relationship between FM and burnout. These findings may be helpful in understanding the psychological status and distress of Chinese medical workers, and in developing effective intervention measures to reduce burnout symptom and defensive medical practice in China.

## Data Availability

The datasets used and analyzed during the current study are available from the corresponding author on reasonable request.

## Abbreviations

FM: Fear of malpractice
LC: Legal Consciousness Scale
EE: Emotional exhaustion
DP: Depersonalization
PA: Personal accomplishment
MBI: Maslach Burnout Inventory
MBI-HSS: Maslach Burnout Inventory-Human Service Survey
JD-R: Job Demands-Resources
EFA: Exploratory factor analysis
CFA: Confirmatory factor analysis
CFI: Comparative Fit Index
TLI: Tucker Lewis Index
SRMR: Standardized Root Mean Square Residual
RMSEA: Root Mean Square Error of Approximation
ANOVA: A one-way analysis of variance
